# Relationship between Physical Activity and Physical Fitness in Preschool Children: A Cross-Sectional Study

**DOI:** 10.1155/2017/9314026

**Published:** 2017-11-21

**Authors:** Hui Fang, Minghui Quan, Tang Zhou, Shunli Sun, Jiayi Zhang, Hanbin Zhang, Zhenbo Cao, Guanggao Zhao, Ru Wang, Peijie Chen

**Affiliations:** ^1^School of Kinesiology, Shanghai University of Sport, Shanghai 200438, China; ^2^College of Youth, Shanghai Normal University, Shanghai 200083, China; ^3^Health Promotion Center, Zhejiang Provincial People's Hospital, Hangzhou, Zhejiang 310014, China; ^4^Department of Physical Education, Nanchang University, Nanchang, Jiangxi 330031, China

## Abstract

**Purpose:**

To evaluate the relationships between objectively measured physical activity and physical fitness among preschool children.

**Methods:**

A total of 346 participants (201 boys and 145 girls) aged 3.5–5.5 years (M = 4.5 yr, SD = 0.47) from Shanghai, China, completed physical fitness assessments, including triceps skinfold thickness (TSFT), grip strength, tennis throwing, sit and reach test, standing long jump, balance beam, 10mSRT, and 20mSRT. Physical activity was objectively measured by ActiGraphGT3X+ accelerometer. Multiple linear regression models were used to explore the cross-sectional associations between PA and physical fitness after adjusting for age, gender, BMI, and valid wearing time.

**Results:**

Positive associations were observed between stand long jump (*p* < .01), tennis throwing (*p* < .01), laps in 20mSRT (*p* < .01), and MVPA. However, TSFT (*p* < .05), time in 10mSRT (*p* < .01), and balance beam (*p* < .05) were negatively associated with MVPA. Furthermore, positive associations were found between stand long jump (*p* < .01), tennis throwing (*p* < .01), and MVPA only in boys. Negative associations were found between time on balance beam (*p* < .01) and MVPA only in girls.

**Conclusions:**

MVPA appears to be an effective and reliable predictor of preschoolers' physical fitness. Boys' body composition, muscular strength, explosive strength, agility, aerobic fitness, girls' agility, aerobic fitness, and balance could improve as MVPA increases.

## 1. Introduction

Children are generally regarded as the most active population. However, given the prevalence of adiposity, inactivity in preschool children has presented a major public health concern over the past few decades [1, 2]. Physical fitness is considered an important element in preventing childhood obesity. Obesity prevention can be achieved by the recognition of its relationship with physical activity (PA) habits, health, and welfare [3]. Physical fitness in early childhood is a powerful marker of health [4] and can be grouped into two broad categories: health-related fitness (aerobic fitness, muscular strength, muscular endurance, and flexibility) and skill-related fitness (agility, balance, coordination, power, reaction time, and speed) [5]. Numerous studies [6–9] have reported that preschool children's physical fitness is declining at an alarming rate and that their PA is far from achieving the International PA Guidelines, like the National Association for Sport and Physical Education (NASPE). Urbanization process, security consideration, environment pollution, public sports area accessibility, and changes in family structure seem to be factors involved in this observed decline in PA. Some data indicates that children are losing the metabolic effect of fitness that might protect them from excessive weight gain and metabolic diseases [10]. On the contrary, PA levels are tracked from childhood to adolescence and from adolescence to adulthood, with moderate to strong coefficients for cardiorespiratory fitness and strength in addition to PA and obesity [11, 12]. These findings suggest that poor physical fitness, inactive PA, and high cardiovascular disease risk are all tightly associated.

Adequate physical activities are significant bases for physical fitness in adolescents and young adults. Higher levels of PA, especially moderate-to-vigorous physical activity (MVPA), are significantly associated with improved fitness, such as body composition optimization, enhanced bone health, cardiopulmonary function improvement, and adiposity prevention in adolescents [13]. Recently, there have been studies focused on the relationship between fitness and PA levels in youth and school-aged children [10, 13, 14]. From the perspective of life-span development, early childhood is a critical period to promote and establish positive health behaviors, with levels of PA and physical fitness status tracking from early childhood to adolescence, and will continuously reap lifelong benefit [15].

Early years provide a window of opportunity for children to develop fundamental motor skills (FMS). These skills are considered the building blocks of more complex and specialized movements. In preschool children, a new model has recently suggested a reciprocal and developmentally dynamic relationship between PA and motor skill competence, which could possibly be mediated by physical fitness to a significant degree [16], while other studies have described that PA is positively, although weakly, related to different motor skills in school and preschool children [10, 17, 18]. Additionally, longitudinal studies have revealed that PA is positively associated with both motor skill and aerobic fitness at baseline and after longitudinal changes. Specifically, only vigorous physical activity (VPA), not total physical activity (TPA) or moderate physical activity (MPA), was related to changes in aerobic fitness. Higher PA was associated with less % body fat at baseline, but not with its changes over time. Baseline PA (rather than the subsequently increased PA) was an important determinant of aerobic fitness, indicating that the relationship between physical fitness and PA may be more interrelated in preschoolers than adolescents [10]. This relationship may be explained by preschoolers' dynamic growth and development. These findings inferred the benefits of being active from early childhood. Children's fitness levels change in concert with habitual levels of PA, but the directionality of this relationship is unclear [19]. Low PA may be an underlying causes of declines in fitness [20]. Meanwhile, understanding the profound relationship between PA behaviors and physical fitness in early childhood is essential in the development of effective interventions for this group.

However, to our knowledge, little is known about the relationship between PA and physical fitness in preschool Chinese children. Therefore, the purpose of the current study was to comprehensively investigate the cross-sectional relationship between objectively measured PA and physical fitness in a sample of preschool children in Shanghai.

## 2. Methods

### 2.1. Participants

Ten kindergartens located in Yangpu and Baoshan districts of Shanghai were randomly selected and recruited to participate in the study between August 2013 and November 2014. After receiving the approval from the directors of 8 kindergartens (2 kindergartens withdrew), parents' meetings were orderly organized in each kindergarten. Inclusion criteria for the participants include (1) child in the 2nd grade in kindergarten; (2) physical health and no limitations in exercise participation; (3) informed consent signed by the parent/legal guardian of the child. All the potential participants were comprehensively informed of the aims and whole process of the study and 374 signed written informed consent forms from parents or legal representatives were obtained (88% response rate). 28 participants who had known medical conditions that could affect motor proficiency or participation in PA were eliminated. Thus, a total of 346 3.5–5.5-year-old children (201 boys and 145 girls) were included. The study was approved by the Institution Review Board from the Ethics Advisory Committee of Shanghai University of Sport.

### 2.2. Measures and Procedures

#### 2.2.1. Measurement of Physical Activity

PA was assessed over 7 consecutive days (5 weekdays and weekend days) by the ActiGraph GT3X+ accelerometer (Actigraph LLC, Pensacola, FL, USA) which has been widely used for objective PA measurement. Participants were allowed to remove the accelerometer while sleeping, showering, or swimming. Parents were instructed to help their children wear the accelerometer above the iliac crest of the right hip by an adjustable elastic belt. For data to be considered valid, at least 3 days of recording (2 weekdays and 1 weekend day) with a minimum of 480 minutes of wear time per day was required. Sequences of at least 20 min of consecutive zero values were removed and interpreted as “accelerometer not worn.” Raw accelerometer data was downloaded and converted into activity counts per second (epoch) by using the ActiGraph propriety software (ActiLife Version 6.11.5). TPA, LPA, and MVPA were based on cutoffs published by Pate et al. [21] (100–1679 counts/min for LPA, 1680–3367 counts/min for MPA, and ≥3368 counts/min for VPA). Each second count over the specific cutoff was summarized in the corresponding intensity level group.

#### 2.2.2. Measurement of Physical Fitness

Physical fitness measurements were conducted in preschool settings by trained research assistants according to the National Physical Fitness Measurement Standards Manual (preschool children version) [22]. In total, eight categories of physical fitness tests were chosen for our study (five tests from the National Physical Fitness Measurement Standards Manual and three additional tests). The TSFT, grip strength, tennis throwing, sit and reach test, and 20mSRT were chosen as they represent health-related fitness (body composition, muscular strength, flexibility, and aerobic fitness). Meanwhile, the 10mSRT, standing long jump, and balance beam were selected because they represent some aspects of skill-related physical fitness (agility, explosive strength, and balance).

*Body Fat*. Each participant's triceps skinfold thickness (TSFT) was measured using a caliper, and scores were used to represent body fat percentage. TSFT (mm) was measured by highly trained assistants following recommended protocols. Skinfold thickness measurements were performed by lifting a fold of skin and subcutaneous fat away from the underlying muscle and bone. The triceps skinfold was lifted parallel to the long axis of the body, midway on the back of the hanging freely upper arm. All measurements were taken on the right side of the body.

*Muscular Strength*. Muscular strength was assessed by two aspects: grip strength and upper limb and lumbar abdomen strength through two individual tests. Grip strength was measured by a dynamometer twice for each child using their right hand in a standing position, per standardized protocol. We used only the maximum values (kilograms) obtained from participants' dominant right hands. Upper limb and lumbar abdomen strength were measured by the tennis throwing test. Children were required to stand behind the baseline and throw tennis ball as far as possible two times. The maximum distance (m) was recorded for each trial.

*Flexibility*. Flexibility was assessed by the sit and reach test. The participants sat on the floor barefoot, with their legs together, knees fully extended, and soles of the feet pressed against the edge of the Flex-Tester box. Participants then were asked to bend forward slowly and use their arms to push the edge of a moveable board as far as possible without bending at the knees. The task was repeated twice, and the distance the board was shifted was recorded for trial.

*Aerobic Fitness*. Aerobic fitness was assessed by a multistage 20mSRT [23]. The test measures aerobic capacity by running back and forth over 20 m with an initial running speed of 8.0 km/h. The progressive 0.5 km/h increase in running speed every minute was indicated by a sound. Maximum performance was determined when the child could no longer maintain the pace or when the child decided to stop due to exhaustion. The 20mSRT has been found to be a reliable (test retest *r* = 0.73–0.93) [23] and valid measure of maximum oxygen consumption as measured by treadmill testing (*r* = 0.69–0.87) [24]. Due to the very young age of the children, some formal adaptations of the original test were made by having an experienced research assistant running with the children until the end of the test to provide adequate pace. The test results were expressed as stages (laps).

*Agility*. Agility was assessed by the 10mSRT. Participants ran 10 m from the starting point, bypassing the retracing point, and ran back to the starting point as fast as possible. The test was measured in seconds. Each child had two attempts and the faster one was used for further data analysis.

*Explosive Strength*. Explosive strength was measured by the standing long jump test. Participants were asked to swing their arms and jump forward with full strength for two individual attempts. The maximum distance (cm) was recorded.

*Balance*. Dynamic balance was tested by balancing barefoot on a balance beam (3 m long, 10 cm wide, and 30 cm high). Children were required to stand on the beam, raise both arms to their sides, and walk as fast as possible from one end to the other. If a child fell midway on the beam, remeasurement was required. The shortest travel time was recorded using a chronograph.

### 2.3. Statistical Analyses

All data were analyzed using SPSS Statistics Version 22.0. (IBM Corporation, USA). For descriptive analyses, the results are presented as means ± SD. Gender differences in age, BMI, PA, and fitness variables were analyzed using independent sample *t*-test. To assess the relationships between PA and physical fitness, we used multiple linear regression models with physical fitness as dependent variable and PA as independent variables, while controlling for age, BMI, gender, and valid wearing time. The significance level was set at 0.05 for all statistical analyses.

## 3. Results

Characteristics of the 346 participants are shown in Table 1. The sample was 58.1% boys and the mean age of participants was 4.56 ± 0.47 years. There are significant gender differences in BMI (*t*(334) = 3.219, *p* < .01), LPA (*t*(301) = 2.240, *p* < .05), MVPA (*t*(301) = 2.204, *p* < .05), and TPA (*t*(301) = 2.608, *p* < .01). On average, children engaged in TPA for 167.99 ± 35.07 min per day, including 97.17 ± 17.54 min of LPA and 70.82 ± 17.53 min of MVPA. Furthermore, there were significant gender differences between LPA, MVPA, and TPA. Compared with girls, boys were more physically active and engaged in significantly more LPA (99.10 ± 18.51 min per day), MVPA (72.66 ± 18.95 min per day), and TPA (171.76 ± 37.64 min per day).

Grip strength (*t*(333) = 2.935, *p* < .01), tennis throwing (*t*(333) = 5.040, *p* < .01), and sit and reach test (*t*(331) = −4.238, *p* < .01) significantly differed between genders. This finding indicates that boys had greater muscular strength than girls, while the latter show flexibility than the former during the early childhood period. No significant gender difference was found in TSFT, standing long jump, 10mSRT, 20mSRT, or balance beam test (all *p* > .05).

Differential associations between physical fitness and PA are detailed in Table 2 using multiple linear regression analyses. Overall, there were significant associations between physical fitness and MVPA (and LPA) in preschool children after adjusting for age, BMI, gender, valid wearing time, and potential clustering of preschools. Specifically, the stand long jump (*R*^2^ = 0.194, *p* < .01), tennis throwing (*R*^2^ = 0.202, *p* < .01), and laps in the 20 m shuttle (*R*^2^ = 0.207, *p* < .01) were all positively associated with MVPA, while triceps skinfold thickness (*R*^2^ = 0.543, *p* < .05), time in the 10mSRT (*R*^2^ = 0.275, *p* < .01), and time on the balance beam (*R*^2^ = 0.043, *p* < .05) were all negatively associated with MVPA. Also, positive associations were noted between tennis throwing (*R*^2^ = 0.202, *p* < .05), laps in the 20 m shuttle (*R*^2^ = 0.207, *p* < .05), and LPA; negative associations were noted between time in the 10mSRT (*R*^2^ = 0.275, *p* < .05), time on the balance beam (*R*^2^ = 0.043, *p* < .05), and LPA. However, no significant associations were found between the grip strength and sit and reach test or MVPA and LPA in our study.

Tables 3 and 4 showed the cross-sectional associations between eight aspects of physical fitness and PA in boys and girls, respectively, using multiple linear regression analyses after adjusting for age, BMI, valid wearing time, and potential clustering of preschools. Overall, agility (time in the 10mSRT) and aerobic fitness (laps in the 20mSRT) were significantly associated with MVPA in both genders (*R*^2^ = 0.269,* R*^2^ = 0.302, and *p* < .01;* R*^2^ = 0.208,* R*^2^ = 0.193, and *p* < .01). Positive associations were found between the standing long jump (*R*^2^ = 0.235, *p* < .01), tennis throwing (*R*^2^ = 0.155, *p* < .01), and MVPA; negative associations were found between triceps skinfold thickness (*R*^2^ = 0.526, *p* < .05) and MVPA only in preschool boys. Negative associations were noted between time on balance beam (*R*^2^ = 0.064, *p* < .01) and MVPA only in preschool girls.

In addition, significant positive associations were also found between the standing long jump (*R*^2^ = 0.235, *p* < .05) and LPA in preschool boys, but not in girls. Regardless of gender, flexibility (sit and reach) and muscular strength (grip strength) showed no association with PA (MVPA or LPA).

## 4. Discussion

Preschool children are always subject to dynamic physical growth and development. Due to the significant impacts of PA on the health of preschool children, an accurate understanding of the relationship between PA and physical fitness is of great importance. The current study explored cross-sectional associations between physical fitness and PA in Shanghai preschool children. To our understanding, this is the first study investigating the relationship between physical fitness and PA in Shanghai preschoolers. These findings may help provide reference data for this population. Our results are partly in concordance with previous cross-sectional studies in preschoolers [25–27], which indicated that PA was sex- and age-adjusted, and positively associated with agility, balance, and aerobic fitness. Physical fitness among normal-weight preschool children was significantly better in comparison to their overweight counterparts [3]. A recent study reported that obese and overweight children were less physically active and had lower physical fitness than normal-weight children [28]. Our results also showed that MVPA appeared to be an effective predictor of preschool children's physical fitness regardless of gender. Furthermore, we noticed a gender difference in the relationship between physical fitness and PA level. Boys' body fat, upper limb muscular strength, explosive strength, agility, and aerobic fitness may have improved as they engaged in more MVPA (or LPA). For girls, MVPA showed significant associations with balance, agility, and aerobic fitness. Meanwhile, compared to MVPA, LPA seemed to do relatively little in improving physical fitness (except agility and boys' explosive strength) during the early childhood period of our study.

The current study adds important information regarding the broader relationship between PA and different aspects of physical fitness in preschool-aged children. Few studies in this age range have been conducted and their results are inconsistent. The reasons for the discordant results among those studies, in part, may include sample size, age, study design, physical fitness, PA measurement, and other confounding variables such as lifestyles. Many previous studies have only focused on one or two aspects of physical fitness (i.e., aerobic fitness or muscular strength) and its relationship between PA in children. Several studies [4, 29–31] reported a stronger relationship between VPA and a higher aerobic fitness level in school children and adolescents. However, in Bürgi et al.'s study [10], no significant association between VPA and higher aerobic fitness was observed. In our study, MVPA was associated with TSFT, especially in boys. As TSFT was always used to represent the % body fat, our results supported the opinion that high levels of PA were probably protective against childhood obesity. Furthermore, a recent study has demonstrated that an additional 10 min/day in MPA or VPA increased the bone stiffness by 1-2% on average, and muscle strength did not affect the associations of PA and bone stiffness based on a large sample of 2–10-year-old children [32]. Unfortunately, the relationship between muscular fitness and PA was not examined in this study, and all the physical fitness tests were administered to school children, not preschool children.

Other studies focused on the relationship between fundamental movement skills (both locomotor and manipulative skills like running, throwing, catching, sliding, and galloping) and PA in preschool period [8, 33, 34]. On one hand, even though there has been strong evidence for a significant relationship between them, we still consider them as two separate notions. Physical fitness is basic element of FMS, especially during the early childhood period, yet few studies have involved FMS in their profound relationship in this area. On the other hand, children with higher levels of FMS competence will seek to participate in PA and sports, while failure to master such skills will result in self-selected withdrawal from participation. Performance level in motor skills may predict PA to some extent. However, our study suggests that the relationship between PA and physical fitness is at least partially dominated by the impact of PA on physical fitness. PA may drive development and improve physical fitness status in preschoolers [10]. We believe that higher physical fitness does not guarantee a more active lifestyle, but that it is essential to continuously promote PA throughout childhood to obtain a child's physical wellbeing.

In addition, no significant association was observed in our study between either flexibility or grip strength and PA (either MVPA or LPA) in both genders. Although flexibility is also an indispensable component of physical fitness, the existing literature described the association between PA and flexibility as “surprisingly limited” and only focused on older adults [35]. A longitudinal study indicated adolescents' flexibility, endurance strength, and PA were predictors of adult neck tension, lower-back pain, and knee injury. However, the relationship between them was not conducted [36]. Some possible explanations for these findings may be that the positive relation between the aforementioned values may exist within a certain sensitive period, that it has recently emerged and is still very weak, or that it might strengthen over time. As our current cross-sectional study only focused on this particular age, it may lead to incomplete conclusions.

An important strength of the current study is that we investigated the relationship between physical fitness and PA in preschoolers in a holistic manner rather than only focusing on certain physical fitness elements. Further strengths are the comprehensive assessments of sensitive process-based measures of physical fitness, objective measurement of PA in a young population, and adjustments of all analyses for potential confounders. Moreover, we have considered the gender difference in the relationship between certain physical fitness tests and PA in preschoolers.

Nevertheless, our study limitation is that physical fitness measurements were conducted in the preschool settings, which may not be accurate against laboratory tests in this epidemiological approach. Fitness tests in more experimental settings may better reflect performances levels than in real life. In addition, in this particular age group, children may still lack understanding and coordination during the fitness tests. Another limitation is that we only used the TSFT variable instead of the body fat percentage, and there was small percent of obese children in our study. Further studies are warranted to detect a profound relationship between body composition and PA within this age group. Moreover, our sample is recruited mainly from two districts of Shanghai, which may not represent the whole preschool population and limits the generalizability of the findings.

## 5. Conclusion and Clinical and Public Health Implications

In conclusion, our study provides novel information regarding the relationship between physical fitness and PA in 3.5–5.5-year-old Chinese preschool children. Based on our results, PA correlates with physical fitness aspects including upper limb muscular strength, explosive strength, agility, balance, and aerobic fitness. We observed pronounced association with more intense physical activities in our study, and MVPA appears to be an effective and reliable predictor of preschool children's physical fitness. Thus we suggest that MVPA is essential for the development and promotion of physical fitness in preschoolers, especially with regard to body fat, explosive strength, balance, agility, and aerobic fitness. Furthermore, certain aspects of physical fitness status may have improved, respectively (boys' body composition, muscular strength, explosive strength, agility, and aerobic fitness; girls' balance, agility, and aerobic fitness), if they engage in more MVPA. Great importance should be placed, and different interventions may be taken as gender difference still exists. These results are important to consider when establishing gender-specific PA recommendations or targeting health promotion interventions during early childhood. The findings of this study will inform the development of health promotion strategies for researchers and educational policymakers by targeting preschool children who are at risk of physical inactivity, promoting their physical fitness and wellbeing in a holistic manner.

## Figures and Tables

**Table 1:** Descriptive statistics for age, BMI, physical fitness, PA, and gender difference.

	Group (*n* = 346)		Boys (*n* = 201)		Girls (*n* = 145)		Gender difference (*p*)
	Mean	SD	Mean	SD	Mean	SD	
Age (year)	4.56	0.47	4.58	0.46	4.52	0.48	>.05
BMI (kg/m^2^)	16.20	1.80	16.47	1.84	15.84	1.69	<.01
PA							
LPA (min/day)	97.17	17.54	99.10	18.51	94.56	15.85	<.05
MVPA (min/day)	70.82	17.53	72.66	18.95	68.34	15.13	<.05
TPA (min/day)	167.99	35.07	171.76	37.46	162.90	30.98	<.01
Physical fitness							
Triceps skinfold thickness (mm)	10.18	2.78	10.20	3.02	10.15	2.43	>.05
Grip strength (kg)	6.73	2.32	7.04	2.35	6.30	2.21	<.01
Standing long jump (m)	83.43	16.86	84.76	16.92	81.60	16.66	>.05
Tennis throwing (m)	4.76	1.52	5.10	1.66	4.29	1.16	<.01
Sit and reach (cm)	10.34	4.68	9.43	4.51	11.58	4.65	<.01
10 m shuttle (s)	7.23	0.86	7.20	0.92	7.29	0.78	>.05
20 m shuttle (lap)	12.18	4.49	11.90	4.27	12.55	4.67	>.05
Balance beam (s)	14.83	15.13	15.26	17.13	14.26	12.01	>.05

**(a):** 

	Triceps skinfold thickness (mm)			Grip strength (kg)			Stand long jump (cm)			Tennis throwing (m)		
	*β*	*R* ^2^	*p*	*β*	*R* ^2^	*p*	*β*	*R* ^2^	*p*	*β*	*R* ^2^	*p*
PA		0.543			0.217			0.194			0.202	
LPA	0.014		>.05	0.029		>.05	0.097		>.05	0.187		<.05
MVPA	−0.181		<.05	0.084		>.05	0.348		<.01	0.316		<.01

**(b):** 

	Sit and reach (cm)			10 m shuttle run (s)			20 m shuttle run (lap)			Balance beam (s)		
	*β*	*R* ^2^	*p*	*β*	*R* ^2^	*p*	*β*	*R* ^2^	*p*	*β*	*R* ^2^	*p*
PA		0.051			0.275			0.207			0.043	
LPA	0.037		>.05	−0.183		<.05	0.201		<.05	−0.181		<.05
MVPA	−0.098		>.05	−0.485		<.01	0.378		<.01	−0.196		<.05

Adjusted for age, BMI, gender, valid wearing time, and potential clustering of preschools.

**(a):** 

	Triceps skinfold thickness (mm)			Grip strength (kg)			Stand long jump (cn)			Tennis throwing (m)		
	*β*	*R* ^2^	*p*	*β*	*R* ^2^	*p*	*β*	*R* ^2^	*p*	*β*	*R* ^2^	*p*
PA		0.526			0.197			0.235			0.155	
LPA	0.026		>.05	0.078		>.05	0.283		<.05	0.188		>.05
MVPA	−0.195		<.05	0.109		>.05	0.469		<.01	0.407		<.01

**(b):** 

	Sit and reach (cm)			10 m shuttle run (s)			20 m shuttle run (lap)			Balance beam (s)		
	*β*	*R* ^2^	*p*	*β*	*R* ^2^	*p*	*β*	*R* ^2^	*p*	*β*	*R* ^2^	*p*
PA		0.025			0.269			0.208			0.048	
LPA	0.106		>.05	0.228		<.05	0.141		>.05	0.187		>.05
MVPA	0.019		>.05	−0.513		<.01	0.344		<.01	−0.134		>.05

Adjusted for age, BMI, valid wearing time, and potential clustering of preschools.

**(a):** 

	Triceps skinfold thickness (mm)			Grip strength (kg)			Stand long jump (cn)			Tennis throwing (m)		
	*β*	*R* ^2^	*p*	*β*	*R* ^2^	*p*	*β*	*R* ^2^	*p*	*β*	*R* ^2^	*p*
PA		0.568			0.180			0.099			0.089	
LPA	−0.077		>.05	0.042		>.05	0.032		>.05	0.164		>.05
MVPA	−0.041		>.05	0.151		>.05	0.156		>.05	0.148		>.05

**(b):** 

	Sit and reach (cm)			10 m shuttle run (s)			20 m shuttle run (lap)			Balance beam (s)		
	*β*	*R* ^2^	*p*	*β*	*R* ^2^	*p*	*β*	*R* ^2^	*p*	*β*	*R* ^2^	*p*
PA		0.070			0.302			0.193			0.064	
LPA	0.075		>.05	−0.234		<.05	0.142		>.05	0.077		>.05
MVPA	0.172		>.05	−0.465		<.01	0.423		<.01	−0.321		<.01

Adjusted for age, BMI, valid wearing time, and potential clustering of preschools.
